# Proteomic Profiling Towards a Better Understanding of Genetic Based Muscular Diseases: The Current Picture and a Look to the Future

**DOI:** 10.3390/biom15010130

**Published:** 2025-01-15

**Authors:** Marc Pauper, Andreas Hentschel, Malte Tiburcy, Sergi Beltran, Tobias Ruck, Ulrike Schara-Schmidt, Andreas Roos

**Affiliations:** 1 Centro Nacional de Análisis Genómico (CNAG), Baldiri Reixac 4, 08028 Barcelona, Spain; marc.pauper@cnag.eu (M.P.); sergi.beltran@cnag.eu (S.B.); 2 Departament de Genètica, Microbiologia i Estadística, Facultat de Biologia, Universitat de Barcelona (UB), 08028 Barcelona, Spain; 3 Leibniz-Institut für Analytische Wissenschaften-ISAS-e.V., 44227 Dortmund, Germany; andreas.hentschel@isas.de; 4 Institute of Pharmacology and Toxicology, University Medical Center Göttingen, Georg August University, 37075 Göttingen, Germany; m.tiburcy@med.uni-goettingen.de; 5 ZHK (German Centre for Cardiovascular Research), Partner Site Göttingen, 37075 Göttingen, Germany; 6 Universitat Pompeu Fabra (UPF), 08002 Barcelona, Spain; 7 Department of Neurology, Medical Faculty and University Hospital Düsseldorf, Heinrich Heine University, 40225 Düsseldorf, Germany; tobias.ruck@med.uni-duesseldorf.de; 8 Department of Neurology, BG-University Hospital Bergmannsheil, Ruhr University Bochum, 44789 Bochum, Germany; 9 Heimer Institute for Muscle Research, BG-University Hospital Bergmannsheil, 44789 Bochum, Germany; 10 Department of Pediatric Neurology, Centre for Neuromuscular Disorders, University Duisburg-Essen, 45147 Essen, Germany; ulrike.schara@uk-essen.de; 11 Brain and Mind Research Institute, Children’s Hospital of Eastern Ontario Research Institute, Ottawa, ON K1H 8L1, Canada

**Keywords:** muscle proteomics, muscle biochemistry, muscle organoids, neuromuscular disease biomarker, white blood cells in muscle disease, muscle laser capture microdissection

## Abstract

Proteomics accelerates diagnosis and research of muscular diseases by enabling the robust analysis of proteins relevant for the manifestation of neuromuscular diseases in the following aspects: (i) evaluation of the effect of genetic variants on the corresponding protein, (ii) prediction of the underlying genetic defect based on the proteomic signature of muscle biopsies, (iii) analysis of pathophysiologies underlying different entities of muscular diseases, key for the definition of new intervention concepts, and (iv) patient stratification according to biochemical fingerprints as well as (v) monitoring the success of therapeutic interventions. This review presents—also through exemplary case studies—the various advantages of mass proteomics in the investigation of genetic muscle diseases, discusses technical limitations, and provides an outlook on possible future application concepts. Hence, proteomics is an excellent large-scale analytical tool for the diagnostic workup of (hereditary) muscle diseases and warrants systematic profiling of underlying pathophysiological processes. The steady development may allow to overcome existing limitations including a quenched dynamic range and quantification of different protein isoforms. Future directions may include targeted proteomics in diagnostic settings using not only muscle biopsies but also liquid biopsies to address the need for minimally invasive procedures.

## 1. Introduction

**Figure 1:**
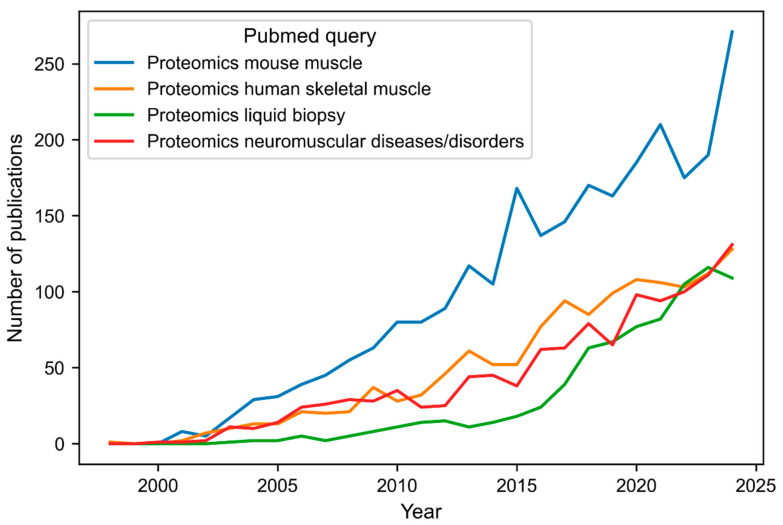
Line plot highlighting the upward trend in the number of publications for three search queries related to proteomics.

Skeletal muscle constitutes 40% of the human body mass and plays vital roles in locomotion and whole-body metabolism. As a major regulatory tissue of whole-body metabolism, skeletal muscle is composed of a diverse mixture of muscle fiber types: type I (slow oxidative), type IIa (fast oxidative), and type IIx (fast glycolytic) [1]. Aging and several (hereditary) diseases differentially affect the various fiber types, and therefore, investigating the changes in the proteome in a fiber-type-specific manner is essential [2]. Over the last decades, protein analytics represented an important aspect in the diagnostic workup of patients clinically presenting with muscular diseases, for which more than 200 different genetic subtypes have been described [3]. For that purpose, immunoblot studies on protein extracts of muscle biopsies were carried out in addition to immunostaining studies and histological studies on muscle biopsy specimens. Although this analytical approach enabled us to obtain hints toward underlying genetic defects, only small panels of proteins could be examined at once. This, in turn, represents a considerable limitation given the growing list of genes and auto-immune defects linked to the manifestation of muscular disease and might even result in a diagnostic odyssey for patients, challenging proper genetic counseling [3]. Not only does the amount of material required for a very comprehensive biochemical profiling of skeletal muscle biopsies pose a limitation, especially in the field of neuropediatrics, but also the availability of suitable antibodies along with the time and costs associated. In light of these facts, it is obvious that alternative analytical approaches such as proteomics may provide the potential to overcome these limitations by enabling the high-throughput study of thousands of proteins in a single experiment while requiring a minimal amount of material. Accordingly, proteomic-based analysis of proteins has made enormous inroads into the research of muscle diseases, especially in the past decade. This was essentially aimed at improving the understanding of underlying pathophysiologies, which is an important prerequisite to the identification of starting points for therapeutic intervention concepts. The investigation of many of the individual entities of muscle diseases was based on the use of biomaterial from either animal models (e.g., mouse models and zebrafish) patients, or both. These aspects are mirrored by the increase of respective publications listed in Pubmed as visualized in Figure 1. Studies on animal models also allowed us to determine the proteomic signature of different muscle fiber types that are present in the complex muscle tissue. For instance, an in-depth study on laser-microdissected mouse muscle fibers immunolabeled for Myosin heavy chain isoform investigated by mass spectrometry revealed unique fiber type protein profiles, confirming fiber-type-specific metabolic properties and indicating a more versatile function of type IIx fibers. Of note, the same study highlighted that multiple myopathy-associated proteins were enriched in type I and IIa fibers [4]. A comparative proteomic and transcriptomic study published recently enabled the molecular dissection of skeletal muscle in infants and adults thus enabling biochemical insights into healthy muscle aging [5].

Moreover, the determination of the overall protein composition of cultured muscle cells by proteomic profiling enabled the classification of immortalized human muscle cell lines as a suitable in vitro system for the study of (genetic) muscle diseases [6,7,8,9]. Along this line, the proteomics-based study of primary cells cultured from skeletal muscle biopsies collected from patients is a promising analytical approach to decipher pathophysiologies of inherited muscle diseases or to unveil liquid biomarkers indicative of the associated myopathology, as exemplified for DUX4 targets in the context of Facioscapulohumeral muscular dystrophy (FSHD) [10,11]. However, the use of patient material to examine the underlying pathobiochemistry is often limited by the availability of biopsy material. On the one hand, these are rare, sometimes even nano-rare diseases, and on the other hand, increasingly fewer biopsies are being carried out in the age of next-generation sequencing technologies. Nevertheless, these studies have contributed enormously to improving the understanding of the genesis of muscle diseases, which is due, among other things, to the integration of proteomic data with other omics datasets [5,12,13,14]. In the last 15 years, proteomic approaches have shown enormous and rapid development, which has led to a constant increase in the number of quantifiable proteins. Whereas in the past only a couple of hundred proteins could be robustly quantified using difference gel electrophoresis (DIGE) technology on protein extracts from human skeletal muscles [15], new analytical approaches such as data-independent acquisition (DIA) mode technology offer the possibility of quantifying several thousand proteins [5,16]. This technical development represents an enormous advance in the problem of quenched dynamic range in the quantification of skeletal muscle proteins, which is caused by highly abundant contractile proteins (such as Actin, Myosins, Titin, and Nebulin) that interfere with the detection of other proteins [17].

## 2. Clinical Applications of Proteomics in Muscular Disease

This section explores the clinical applications of proteomics in-depth, outlining the ways proteomic approaches can enhance our understanding of muscular disease mechanisms, aid in diagnosis, and guide therapeutic strategies. We will discuss methodologies that span both “gene-first” and “protein-first” paradigms. In the “gene-first” approach, we evaluate how proteomics analyses can delineate the impact of genetic variants on the corresponding protein structures and functions, providing insight into the disease mechanisms at the molecular level. By contrast, the “protein-first” approach leverages proteomic signatures that may point toward underlying genetic defects, thus enabling diagnosis in cases where the genetic data may be ambiguous. Finally, in the next subsections, we discuss the concepts of the discovery of underlying pathophysiologies and how they can lead to therapeutic intervention strategies, patient stratification by biochemical fingerprints, and the identification of serum biomarkers reflective of muscle dysfunction.

These concepts are illustrated through examples and case studies from our work in the framework of the NMD-GPS project (https://nmd-gps.net), which aims to study the pathobiochemistry in a large cohort of neuromuscular disease patients with different diagnoses. These examples offer concrete instances where proteomic data has aided in clarifying pathophysiological mechanisms, bridging proteomic research, and its practical applications in patient diagnosis and care.

(1) Evaluation of the effect of molecular genetic variants on the corresponding protein

DNA sequencing is a standard procedure in the diagnostic workup of muscular diseases with presumed genetic origin (more than 200 related genes have been described thus far [3]). The interpretation of the biological significance of genetic variants to understand pathological mechanisms, provide genetic counseling, and develop therapeutic interventions is a pivotal step in this process. However, despite the introduction of the 2015 American College of Medical Genetics guidelines [18], the interpretation of variants of unknown significance (VUS) remains challenging and often requires several years to yield conclusive results [19,20,21]. The integration of transcriptomics, proteomics, and genomics data enables a comprehensive investigation of the influence of genetic variants on the abundance of transcript and proteins. While RNA-seq can be used to assess the impact of genetic variants by measuring transcript levels, proteomics offers distinct and complementary advantages by directly quantifying protein abundance [22]. Notably, the correlation between transcript and protein abundance has been previously shown to be often poor, highlighting the impact of post-transcriptional regulation [23]. Furthermore, this approach can be used to assess the pathogenicity of variants, not only due to the abundance of the corresponding protein but also due to the possible dysregulation of (metabolic) pathways in which these proteins are involved. As such, proteomics represents an essential and effective method in the diagnosis of genetic muscular diseases and enables the interpretation of the pathogenicity of VUS, enabling accurate genetic counseling for patients and their families [24,25]. An example of applied proteomics in the diagnostic workup of a neuromuscular patient is presented in Appendix A Box 1.

More generally, and as exemplified based on the case presented in Appendix A Box 1, changes in the protein level of functional or physical interactors may be of significant relevance in the context of missense variants. This is especially true in circumstances in which the amino acid substitutions do not influence the abundance of the protein but may affect proper protein-protein interactions and related biological functions, or when the protein encoded by a mutated gene eludes detection by mass spectrometry. Furthermore, the integration of additional omics data can also be supportive in these situations. For example, if metabolically relevant proteins are affected, the results of metabolic or lipidomic studies can further corroborate and complement the results of proteomic analyses [26].

In addition, proteomic analyses can provide information about the abundance of specific proteoforms or whether genetic variants lead to the expression of truncated proteins. Mapping and visualization of the identified precursor peptides on the corresponding protein sequence and comparison of their intensities can yield valuable insight in this context [27,28,29]. For instance, PrIntMap-R is a tool that provides a user-friendly interface to create such visualizations [30]. This is important not only for diagnostic clarification but also for understanding pathophysiology, as protein truncation can lead to either loss of function or gain of toxic function, the latter often due to aggregation of misfolded protein [31]. A paradigmatic example is presented in Appendix A Box 2. However, truncated protein aggregates might be insoluble by standard lysis buffers, in which case proteomic profiling might instead show a decrease compared to controls. Further proteomic studies on protein aggregate myopathies comparing protein abundances in the general proteome profile with abundances detected in enriched aggregates are crucial to providing biochemical evidence.

(2) Prediction of the underlying molecular genetic defect based on the proteomic signature of muscle biopsies

In addition to the “gene-first” approach where proteomics enables the interpretation of previously identified genetic variants, aberrant protein levels in proteomic studies can instead provide an indication of an underlying genetic defect in a “protein-first” fashion. This has not only been shown to be useful for the identification of new disease-causing genes, such as for FHL1 as the causative factor for Reducing Body Myopathy [32], but is particularly advantageous when gene variants are not detected based on the classic evaluation methods of DNA sequence analysis, where aberrant levels of a certain protein might indicate a causal genetic defect. This was recently described as an HNRNPA1-related proteinopathy with manifestation in childhood [33]. Furthermore, the unbiased identification of dysregulations of various proteins that are functionally related to the protein affected by a genetic defect can also provide an indication of the underlying genetic defect. An exemplary case is presented in Appendix A Box 3.

Even though this analytical strategy may not always pinpoint a specific gene, by identifying a large number of proteins that often exhibit secondary changes in different subtypes, it has the potential to enable a better interpretation of genetic data by detecting pathway-level perturbations [34]. Other prominent examples of this are certain subtypes of limb-girdle muscular dystrophies, such as sarcoglycanopathies, in which the loss of one protein of the complex secondarily causes a loss of the other proteins or even entire subunits [35]. However, to be able to clearly assign these patterns of pathophysiologically-related co-regulations to specific subtypes of muscle diseases, the proteomic-based determination of the precise change in abundance of each component is necessary since different binding partners can be affected to varying extents in the different subtypes. To this end, targeted analyses using stable isotope-labeled peptides would be suitable but must usually be carried out on larger cohorts. The analysis of larger cohorts would be needed to investigate thresholds and thus vulnerabilities of the targeted proteins across different neuromuscular diseases, an important prerequisite to determine pathophysiological dysregulations of clinical relevance. However, this analytical strategy requires special technical equipment and experienced personnel making a rapid implementation into routine diagnostic laboratories challenging.

(3) Uncovering pathophysiologies and patient stratification based on biochemical fingerprints

In addition to the advantages of applied proteomics for the diagnostic workup of patients with hereditary muscle diseases, clinical proteomics also has an enormous potential for exploring starting points for therapeutic intervention concepts based on the identified pathophysiologies. Protein dysregulations that represent affected metabolic pathways can serve as starting points for therapeutic intervention concepts, as demonstrated by the following examples: a representative study aimed to better understand mechanisms of gastrocnemius muscle impairment in peripheral artery disease and unveiled that hypoxia induces accumulation of mitochondria respiratory proteins, reduced activity of rate-limiting glycolytic enzymes, and enhanced integrated stress response that modulates protein translation. These mechanisms may serve as targets for disease modification [36], for instance by strengthening protein folding capacity and/or mitochondrial function and dynamics. Along this line, other proteomic studies also unraveled a profound mitochondrial dysfunction as an effect of a splicing disease manifesting as Myotonic dystrophy type 2 (PROMM—proximal myotonic myopathy) or impaired autophagic flux manifesting as Danon disease [14,37]. Both disease entities are characterized by muscular vacuole myopathy, suggesting that targeting mitochondrial function might represent a promising intervention concept in myopathies associated with vacuoles. Here, the comparison with the proteomic/proteinogenic signatures of other muscle diseases (characterized by the presence of vacuoles; e.g., [38,39]) might allow the definition of common pathomechanisms (such as mitochondrial dysfunction), which can be understood as uniform therapeutic starting points for so-called “basket trials”. Furthermore, these proteomic-based studies demonstrate a common overarching pathomechanism, which in turn may be consistent with the phenotypic grouping. A good example of this is the so-called myofibrillar myopathies, which present with the formation of protein aggregates as a common pathophysiological hallmark. Of note, as unveiled by proteomic studies and subsequent immunofluorescence studies toward validation of mass spectrometry data, these aggregates are mainly composed of structural proteins and chaperones [31,40,41,42]. Here, protein aggregate composition can even be an indication of the respective subtype [40,43,44]. Along this line, proteomic studies also profoundly improved our general understanding of protein aggregate formation in muscle cells by highlighting that a metastable subproteome is present in skeletal muscle. Here, proteomic data suggested that protein solubility is limited in the periphery of the aggregates, in turn resulting in the formation of these pathological inclusions. Of note, while most supersaturated proteins decrease or maintain steady abundance across healthy fibers and inclusion-containing fibers, proteins within the metastable subproteome rise in abundance, suggesting that they escape regulation [44]. Taken together, the results of this study highlight that the supersaturation of a metastable subproteome underlies widespread protein aggregate formation and indeed correlates with the histopathological state of the tissue. The latter is important for the diagnostic evaluation of muscle biopsy specimens.

**Figure A4:**
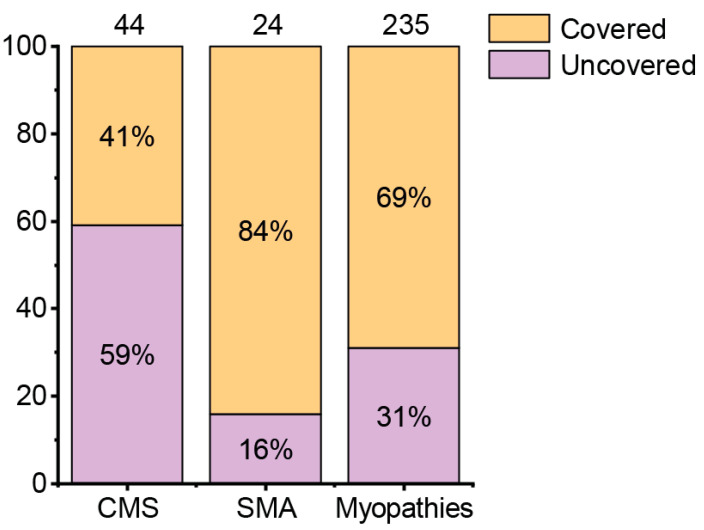
Percentages of coverage of proteins relevant for CMS, SMA, and myopathies in SMO. Numbers above the respective stacked bars refer to the total number of proteins attributed to these diseases based on pathogenic variants in the corresponding genes.

When using patient-derived biopsies, it should be noted that the dissection of primary and secondary pathophysiologies can represent an enormous challenge. This is particularly the case if there is a longer period of time between the manifestation of initial symptoms and the biopsy taking, during which myodegeneration progresses. According to the daily experience in the diagnostic workup of pediatric and adult patients with muscular diseases, the time frame between the first manifestation of clinical symptoms and sample collection may vary. This can for instance be caused by periods of decision-making of the patients or their caregivers. In addition, the first symptoms often only appear after many years of progressive pathobiochemistry and, in this sense, represent the result of years of proteinogenic imbalance in the muscle cells. A prominent example of this is Duchenne muscular dystrophy; proteomic investigation of the skeletal muscles of animal models at the early onset of the disease with subsequent verification on human biopsies may represent a suitable strategy to address this problem but requires that the animal model represents a suitable phenocopy of the human disease. An alternative approach might be the molecular investigation of in vitro systems. The proteomic-based definition of the protein catalog abundant in cultured muscle cells can serve as a criterion for selecting diseases that can primarily be analyzed in these cells in vitro (e.g., [6,7,45]). Of note, our review introduces such a catalog for muscle organoids (Figure A4 and Supplementary Table S1), which are more commonly used to investigate the underlying processes in myopathic diseases.

Proteomic studies of proteins released from muscles by vesicles have also expanded our understanding of the genesis of other diseases. An example of this is the recent discovery of multiple extracellular vesicles secreted from skeletal muscle, traveling through the bloodstream to reach the bone, where they were phagocytized by bone marrow mesenchymal stem/stromal cells (BMSCs). Here, these muscle-derived extracellular vesicles promote osteogenic differentiation of BMSCs and protect against disuse osteoporosis [46]. Another example is the proteomics-based discovery of key proteins promoting re-innervation of muscle fibers facing denervation based on pathologies of the nervous system: a highly sensitive mass spectrometry approach unveiled those proteins involved in myofibrillogenesis are over-represented in target structures, indicating an ongoing process of sarcomere assembly and/or remodeling within this specific area of the muscle fibers toward sarcomeric reassembly [47].

As indicated by the example of nemaline myopathies (outlined above [43]), the determination of proteomic signatures may enable the stratification of patients based on their proteomic fingerprints. While distinct fingerprints in clinically and myopathologically similar patients can open new diagnostic avenues for more precise stratification and subgrouping, similar fingerprints may hint toward shared underlying pathophysiologies. This is exemplified by myopathies associated with defective glycogen metabolism and the resulting build-up of polyglucosan bodies, which were mentioned in the previous section [48,49]. This, in turn, might allow for the grouping of patients according to similar pathophysiological cascades, which could serve as a starting point for unified treatment approaches, such as “basket-trials”. In addition, reliable strategies are needed to classify patients based on their biochemical phenotypes aiming to improve patient selection and treatment outcomes. To address this need, a recent study applied consensus clustering as an unsupervised tool to assign patients suffering from Myasthenia Gravis, a neuromuscular transmission disease, to distinct biological profiles. Here, for in-depth analysis, immunogenomic sequencing was used to study the B cell repertoire of a patient subgroup, and an in vitro assay using primary human muscle cells was used to interrogate serum-induced complement formation. This strategy identified four distinct patient phenotypes based on the proteomic patterns in their serum. Notably, one patient phenotype was characterized by high disease severity and complement activation as defining features, indicating that these patients are more likely to benefit from complement-inhibiting therapies [50].

(4) Proteomics on laser-capture micro-dissected material

Laser capture microdissection (LCMD) enables the selective enrichment of suspicious cells or subcellular structures from muscle biopsies. This technique allows for a detailed analysis of proteomic changes, providing new insights into the molecular mechanisms underlying selective fiber type vulnerability and other pathological processes, such as vacuole formation. In this context, LCMD combined with proteomic analysis has become a valuable method for studying muscular diseases. This approach relies primarily on histological staining of muscle biopsy specimens, revealing aberrant structures, such as protein aggregates and vacuoles. Through LCMD and subsequent proteomic analysis, researchers have been able to determine the protein composition of vacuoles, leading to the discovery of novel genes causative for muscle diseases and the identification of new risk alleles for inflammatory myopathies [32,51,52]. In addition, LCMD and subsequent mass spectrometry-based protein quantification allowed the unveiling of the protein signature of polyglucosan bodies as pathomorphological structures associated with RBCK1 deficiency and impaired glycogen metabolism. This analysis showed that the accumulated proteins primarily belong to glycogen metabolism and protein quality control pathways and that the majority of muscle fibers exhibited glyocogen depletion and redistribution of key enzymes involved in glycogen metabolism to the polyglucosan bodies [48]. Similar findings were obtained for another subtype of myopathy arising from defective glycogen metabolism due to glycogenin-1 deficiency, indicating a common underlying pathophysiology resulting in the build-up of these pathological structures [49]. In contrast to these findings, a study using a similar approach on different pathomorphological structures known as nemaline rods—present in both acquired sporadic late onset nemaline myopathy (SLONM) and inherited nemaline myopathy (iNM)—found differentially expressed proteins between SLONM and iNM [43]. This proteomic finding suggests distinct underlying pathophysiological mechanisms, thereby opening new avenues in patient stratification based on diverging pathobiochemical profiles (see next section).

LCMD combined with mass spectrometry-based proteomics has also proved useful in the investigation of mitochondrial myopathies, which are frequently associated with a mosaic distribution of cytochrome c oxidase+ (COX+) and COX-muscle fibers. A study unraveled that COX+ fibers show higher expression of respiratory chain components and COX-fibers exhibit diverse adaptive responses, including upregulation of mitochondrial ribosomes, translation proteins, and chaperones, revealing compensatory mechanisms in muscle fibers struggling with energy shortage and metabolic stress [53].

(5) Serum biomarkers reflecting muscle dysfunction

Biomarker research represents an important subdiscipline within the broader field of neuromuscular disease research. Findings in this area may open new avenues in patient stratification thereby improving diagnostic management, but also enabling the monitoring of therapeutic intervention concepts, as outlined above. Ideally, these biomarkers should be minimally invasive while still providing robust information on myopathological processes. Although proteomic studies of muscle microdialysates identified potential circulating biomarkers of pathophysiological relevance in Facioscapulohumeral Muscular Dystrophy (FSHD) [54], this approach relies on a relatively invasive sampling procedure compared to the collection of blood samples. As visualized in Figure 1, most research over the last years has consequently focused on surrogate biomarkers of pathophysiological relevance in patient-derived blood samples, resulting in the identification of numerous protein markers that can be monitored in serum and/or plasma of patients with various types of muscular diseases. Most of the marker proteins identified by various proteomic approaches have been described in relation to Duchenne muscular dystrophy (for an overview, see [55]). Here, marker proteins associated with the cytoskeletal apparatus are particularly relevant from a pathophysiological perspective, given the impact that the loss of functional dystrophin has on the cytoskeleton.

However, different analytical approaches were performed also aiming to overcome the problem of highly abundant albumin in the serum samples. Prominent examples are the targeted multiplex enrichment and subsequent quantification of proteins making use of proximity extension assay (Olink) or SOMAscan technologies [11,56,57]. Another promising approach is nanoparticle enrichment mass spectrometry-based proteomics using the Proteograph™ Product Suite workflow (SEER, Inc., Redwood City, CA, USA) [58]. A recent study focusing on the identification of blood biomarkers for Myotonic Dystrophy type 1 (DM1), the most common muscle disease in adulthood, demonstrated that untargeted proteomic profiling on patient-derived primary cells such as fibroblasts enables the pre-definition of pathophysiological relevant proteins which are in turn also differentially abundant in patient-derived serum [59].

Another study has shown that after identifying a blood biomarker, studying its interactome in skeletal muscle cells is a good starting point to investigate its role in the molecular etiology of the disease and accordingly address a pathophysiological role based on ITIH3 as a blood biomarker of pathophysiological relevance at the neuromuscular junction in Myasthenia Gravis [60].

(6) Monitoring of the success of therapeutic interventions

In clinical trials of neuromuscular diseases, response to therapy is often evaluated by correcting protein dysregulations. Proteomic methods are increasingly used to compare the proteomic profile of patients before and during therapy, as exemplified by 5q-related SMA [61,62,63,64]. Blood is the most frequently collected biomaterial from patients, due to its minimally invasive process that can be easily integrated into the study protocols. Consequently, various studies on neuromuscular diseases, such as Myasthenia Gravis, have been conducted [65]. These studies have frequently been performed on animal models thus far and therapeutic success is often assessed by examining protein markers previously discovered by proteomic profiling. These markers can now be measured using accessible analytical techniques, such as Enzyme-linked Immunosorbent Assay (ELISA) [61,66]. Moreover, for a few muscle diseases, proteomic studies have been carried out on muscle biopsies collected from therapy-naive patients, as well as those undergoing therapy. An exemplary study on Pompe disease, a metabolic myopathy, demonstrated that approximately one-third of dysregulated proteins are restored when the enzyme acidic alpha-glucosidase (GAA), missing in the patients due to genetic mutations, is restored, thereby identifying molecular targets to improve patient outcomes [39].

### Limitations and Challenges in the Proteomics-Based Analysis of Muscular Diseases

(1) Analyses of patient-derived biopsies

Usually, muscle biopsies are not collected in the same facility where further processing and analysis are carried out. This implies that the time needed to transport the biopsy from the collection site to the neuromuscular laboratory may vary between different clinics or hospitals. These varying transport times can have an impact on sample quality in institutions or laboratories in which cryopreservation of biopsies is not immediately performed after sample collection. In these scenarios, protein degradation processes and the alteration of post-translational modifications can be triggered, such as phosphorylation. It is worth noting that the biopsies only capture a snapshot of the entire progressive pathophysiology. Accordingly, the extent of (secondary) pathophysiological changes in patient biopsies often varies, which may, in turn, affect the outcomes of proteomic cohort studies. A glaring limitation is the general lack of truly healthy control biopsies, especially when age-matched controls are required, as in neuropediatric diseases [5]. As a result, biopsies that are microscopically classified as normal may need to be used instead.

(2) Quenched dynamic range of muscle proteome

The high dynamic range and abundance of structural proteins in skeletal muscle present significant challenges for high-fidelity proteomics. Skeletal muscle contains some of the largest (e.g., Titin) and smallest (e.g., Phospholamban) proteins in the human proteome. The 10 most abundant proteins, including actin and myosin isoforms, constitute approximately 50% of the total protein mass, while less abundant proteins account for only about 0.1% [67]. This imbalance makes it difficult to detect low-abundance proteins, such as signaling enzymes, during mass spectrometry analysis because fragment ions from abundant proteins can overwhelm the signals from co-eluting peptides of less abundant ones [67]. Moreover, sample preparation is crucial; without adequate fractionation, the presence of highly abundant structural proteins and possible blood contamination can further hinder the identification of low-abundant proteins, thus limiting proteomic depth [68]. Even though DIA provides higher sensitivity compared to DDA [69], these technical factors can still lead to missing values in proteomics quantitative data, particularly for low-abundance peptides that fall below the detection threshold [70]. These missing values are often “missing not at random” (MNAR), as their absence is systematically related to their abundance and the instrument’s sensitivity. This bias complicates downstream statistical analyses and necessitates specialized handling approaches to ensure accurate data interpretation [71].

(3) Proteomic profiling of membrane proteins

Various muscular diseases are caused by genetic variants that encode membrane proteins (MPs), often also affecting other membrane-bound binding partners of the mutated protein. Detecting MPs through mass spectrometry-based protein analyses presents several challenges. First, MPs are often unstable and poorly soluble, as they are sensitive to changes in pH, temperature, and detergents, which can lead to denaturation or aggregation during sample preparation and analysis [72]. Second, the complexity of the protein–membrane system adds further difficulty; MPs are embedded within a lipid bilayer, and their interactions with surrounding lipids are crucial for their function [73]. However, structural proteomics methods require MPs to be isolated from the membrane and separated from lipids to prevent contamination of the analytical high-performance liquid chromatography column and mass spectrometer. Third, the digestion and detection of MPs are hindered by their hydrophobic nature and the scarcity of charged residues that are recognizable by common digestive enzymes [74]. This results in poor protein coverage because reliable identification and quantification with sufficient sequence coverage relies on the generation of numerous unique peptides.

(4) Quantification of protein isoforms

Protein isoforms, which originate from the same gene through mechanisms such as alternative promoter usage or splicing [75] have distinct amino acid sequences and can exhibit different stabilities, molecular binding capabilities, and functional effects [76]. The study of isoform switching is of particular interest in muscular diseases that are associated with altered splicing, such as Myotonic Dystrophy type 1 and 2 [77,78]. However, detecting and accurately annotating these isoforms in mass spectrometry is challenging and highly dependent on the protein database used [79,80]. Although reference databases aim to represent an organism’s proteome, they often fail to account for proteomic variations specific to different tissues, developmental stages, disease states, and individual differences [81]. Discrepancies between the reference database and the actual sample can lead to missed identifications, ambiguous results, or incorrect annotations of protein isoforms. These challenges are particularly significant in the context of neuromuscular diseases, where precise detection and discrimination of isoforms are crucial for understanding disease mechanisms and developing targeted therapies. Prominent examples are diverging missplicing events in Myotonic Dystrophy type 1 and 2 and diverging isoform vulnerabilities in Titinopathies [82,83].

(5) Missing proteins

The human genome contains nearly 20,000 protein-coding genes, and large-scale community efforts have detected evidence of translation for about 90% of them [84,85]. However, alternative splicing and post-translational modifications are responsible for producing a much vaster population of diverse proteoforms [86,87]. The most prominent proteomics approach, bottom-up shotgun proteomics, typically only employs trypsin to digest protein samples prior to analysis by liquid chromatography-tandem mass spectrometry (LC-MS/MS) and relies on a small number of peptides to infer protein identities and quantities. As a result, sequence coverage is generally insufficient to fully characterize the entire proteome of an individual, typically reaching 2000–10,000 identified proteins per sample [88,89,90]. It has been shown that the use of alternative digestion enzymes can improve proteome and phosphoteome coverage [80].

(6) Batch effects

Batch effects pose a significant challenge in large-scale proteomics studies, introducing noise that complicates data interpretation. Such effects can arise from differences in sample preparation, variations in data collection conditions, reagent batches, and changes in technicians or instrumentation. These inconsistencies can cause technical factors to obscure and confound biological signals, raising concerns about their valid interpretation [91]. Additionally, variations in sample aging and environmental factors particularly affect large-scale and longitudinal studies. In large-scale studies, samples may also be collected at different centers, each with its own protocols and storage conditions, further adding to the noise in the dataset [92]. This is especially relevant in the study of rare muscular diseases, where data is often gathered from multiple centers to address the inherent scarcity of samples and data.

The complexity of batch effects in MS-based proteomics is heightened by the technology’s inherent variability. Mass spectrometry results can be influenced by ionization efficiency fluctuations, temperature shifts, and calibration drifts. Liquid chromatography introduces further variability, as column degradation and solvent changes over time can affect results across batches. When experiments are conducted in multiple batches, due to either the study’s size or the available technology, these variations can introduce bias in the combined dataset. Therefore, careful experimental design and proper analysis methodologies are crucial to minimizing batch effects, especially in clinical samples such as muscle or nerve biopsies. Addressing these challenges highlights the need for ongoing research and the refinement of methodologies to ensure the reliability of biological conclusions in MS-based proteomics [92,93].

(7) Computational challenges in proteomics data analysis

Large-scale proteomics experiments generate vast amounts of data that need to be stored, managed, annotated, and analyzed, presenting significant computational challenges. Although targeted DIA has the potential to produce highly robust and reproducible proteomes with greater coverage than DDA [94,95,96], it also introduces specific computational challenges. The simultaneous isolation and fragmentation of precursors in DIA produces complex and multiplexed tandem MS spectra. Analyzing these spectra to accurately identify peptides requires high-quality and extensive spectral libraries. However, generating spectral libraries that fulfill the requirements of comprehensive coverage across experiments using DDA or other experimental methods can be challenging and costly [97]. Library-free approaches, where spectral libraries are generated directly from the DIA data or by predicting spectra of peptide sequences in silico, are computationally intensive and can strongly impact overall peptide and protein identification and quantification [98,99]. In fact, the impact of the choice of computational methods on biological findings has long been recognized [100], but benchmarking, comparing, and selecting the appropriate method remains a complex task [101].

To address these computational challenges, the development and application of efficient, scalable, and accurate computational tools is critical for the advancement of proteomics. Continuous benchmarking of these tools, improvements in spectral library generation methods (both empirical and in silico), and integration of machine learning approaches will likely continue to play a key role in enhancing peptide and protein identification accuracy. Finally, collaborative efforts within the scientific community to share resources, datasets, and standardized workflows are crucial to support not only the reproducibility and robustness of large-scale proteomics studies but also the development of novel methods.

(8) Phosphoproteomics on patient-derived samples

Phosphoproteomics, the large-scale analysis of phosphorylated proteins, is a crucial method for understanding cell signaling and regulatory mechanisms in human tissues, both in health and disease. Mass spectrometry-based investigation of protein phosphorylation warrants meaningful insights into the complexity of protein dysregulations across different pathways. In this context, phosphoproteomics on human samples has already successfully been applied to study the network of exercise-regulated kinases [102]. However, whereas all biopsies can be collected under the same standard operating procedure in research studies, as outlined above, standardized sample collection, and particularly the time elapsed prior to further processing, can pose a challenge in the routine diagnostic workup.

Phosphorylation is a dynamic process extensively used by cells to respond to various perturbations. Consequently, the phosphoproteome is highly sensitive to changes that occur during biopsy procedures, surgical resections, or chemical fixations [103,104]. Previous studies have shown that ischemia can alter up to 1/4 of the phosphoproteome, with significant impacts on phosphotyrosine (pTyr) networks within just 5 min of tissue resection, affecting up to half of the pTyr sites by more than twofold [105]. These rapid changes highlight the importance of immediate tissue preservation to avoid introducing artifacts into the molecular data generated from these samples.

The current gold standard for preserving tissues for phosphoproteomic analyses is snap freezing in liquid nitrogen. However, while this method is effective, immediate snap freezing is often impractical in many clinical settings, such as operating rooms and outpatient clinics. Rapid and proper preservation typically requires a dedicated, trained individual with access to the appropriate materials, which are not always available in these environments. As a result, the application of phosphoproteomics in clinical practice remains very challenging, and the integrity of the phosphoproteomic data can be compromised by delays in sample processing. In contrast, phosphoproteomic studies on diverse murine tissues, including skeletal muscle, have shown to be successful and have expanded the current knowledge of the functional specialization of the mitochondrial phosphoproteome, provided that ideal sampling conditions are met [106].

## 3. Promising Technical Developments and Future Perspectives of Applied Proteomics in Muscular Diseases

(1) DDA versus DIA

Global protein profiling is essential in the research of the molecular etiology of muscle diseases, requiring consistent, accurate, and large-scale protein quantification. Among the commonly used LC-MS/MS-based quantitative proteomics strategies, two targeted approaches, selective/multiple-reaction monitoring (SRM/MRM) and parallel-reaction monitoring (PRM), and two untargeted approaches, DDA and DIA, are widely applied.

Data-dependent acquisition has traditionally been the primary method for untargeted proteomics, where precursor ions are selected for fragmentation based on their intensity. While DDA offers broad protein coverage, it is inherently limited by its stochastic nature, which can result in the inconsistent selection of precursor ions across different runs [107]. This variability leads to issues such as under-sampling, where low-abundance peptides may be missed, and to limited reproducibility, particularly in large sample sets [108]. As a result, DDA can struggle to provide accurate and consistent quantification, making it less ideal for proteomic studies on muscle protein extracts requiring high precision across multiple samples.

In contrast, DIA is a more recently developed technique that addresses many limitations of DDA, combining the strengths of both PRM (high sensitivity and reproducibility) and DDA (broad protein coverage) [109], thus making it particularly suitable for muscle proteomics. In DIA, all ions within a specified range of mass-to-charge ratio are systematically fragmented, capturing a more comprehensive and unbiased dataset that consistently includes peptides of all abundance levels. This leads to the following key advantages over DDA:

• Enhanced reproducibility and quantification accuracy: By fragmenting all ions in a systematic manner, DIA eliminates the variability associated with precursor ion selection in DDA.
• Superior proteome coverage: DIA’s comprehensive data acquisition method allows for the detection of a wider range of peptides, including those that are low in abundance or prone to being missed in DDA.
• Scalability and improved data confidence: Advances in computational tools and software have enhanced the ability to process and analyze DIA data efficiently, making it a robust choice for large-scale proteomics.

Given these advantages, DIA is increasingly recognized as a powerful tool for proteome quantification. Its ability to combine broad protein coverage with high sensitivity, reproducibility, and throughput makes it particularly well-suited for applications in clinical research and personalized medicine, where reliable and comprehensive data are of utmost importance.

(2) Targeted proteomics

While antibody-based techniques such as enzyme-linked immunosorbent assay (ELISA) and immunohistochemistry (IHC) have long been the gold standard for protein quantification in clinical settings, advances in targeted MS are poised to shift this paradigm. Modern high-resolution mass spectrometers have significantly enhanced the capabilities of targeted proteomics, making it an increasingly powerful tool for detecting low-abundance proteins. Targeted MS techniques, such as multiple reaction monitoring (MRM) and parallel reaction monitoring (PRM), have revolutionized quantitative assays for clinically relevant proteins and their modifications, aiding in disease diagnosis, staging, and therapeutic monitoring [110]. Initially developed for protein biomarker verification in plasma and/or serum, such as the discovery of Periostin as a robust blood biomarker for Myotonic Dystrophy type 1 [59], these targeted MS approaches are now increasingly applied in preclinical and biological studies, where they enable robust quantification of peptides and post-translationally modified targets across large sample cohorts [111,112]. Additionally, targeted MS provides high specificity and multiplexing capacity, allowing simultaneous quantification of multiple proteins with high accuracy, an enormous benefit for monitoring biomarker fingerprints in clinical studies or diagnostic-focused patient stratification. In this context, it is important to note that targeted proteomics now often relies on PRM, a high-resolution counterpart to MRM. It offers unparalleled resolution, which is particularly useful in the complex proteomic landscapes typical of clinical samples, where resolving numerous analytes is critical.

Hence, clinical targeted proteomics is rapidly advancing to become an integral part of diagnostic platforms, offering more specific, quantitatively accurate, and potentially cost-effective alternatives to some current gold-standard methods, such as immunoblotting and ELISA. Consequently, targeted proteomics on muscle protein extracts hold the potential to fully replace immunoblot studies in diagnostic settings, enabling the robust quantification of more than 300 neuromuscular proteins while using a minimal amount of biopsy material. In this perspective, targeted proteomics also has the potential to overcome the problem of antibody availability, as specific antibodies for reliable diagnostic quantification of several neuromuscular proteins are still lacking. Another promising clinical application for targeted proteomics is targeted drug monitoring (TDM) [113]. This approach, providing rapid, precise, and robust insights into drug efficacy, could become especially valuable given the growing number of clinical trials for neuromuscular diseases.

(3) MS imaging (MSi)

Understanding the spatial distribution of molecular species within tissues is crucial for elucidating biological processes. Traditional methods like LC-MS often lose this spatial information. In contrast, mass spectrometry imaging (MSi) preserves spatial context by analyzing intact tissue sections, enabling the localization of both endogenous molecules (e.g., metabolites, lipids, proteins) and exogenous compounds (e.g., drugs) within tissue samples. Thus, over the past 2 decades, MSi has become a powerful tool for exploring the spatial distribution of diverse molecules in complex biological systems, with applications in drug discovery, disease evaluation, and proteomics [114]. Advances in technology and data management have solidified MSi as an essential tool in biomedical research. Its workflow parallels traditional histology, facilitating its integration into pathology-related research [115]. In the context of muscular diseases, MSi may help interpret pathological findings obtained on the histological level, leading to better and more comprehensive neuropathological diagnosis. Consequently, MSi is increasingly employed in clinical research and is being integrated into molecular pathology to complement routine histopathological tissue examinations [116,117].

Recent innovations in instrumentation have further strengthened the capabilities of MSi, enabling the detection and characterization of a wide range of biomolecules with unprecedented spatial resolution and sensitivity. MSi can now image potential biomarkers down to cellular resolution and analyze large patient cohorts at speeds that meet clinical demands [114]. This advancement opens new avenues for elucidating biomarkers for muscular diseases, allowing researchers to gain direct insights into their pathophysiological relevance in affected tissues without the need for immunological-based techniques, which might be limited by the availability of suitable antibodies. This also allows for an unparalleled characterization of tissue microenvironments, providing critical insights that link tissue morphology to molecular physiology.

Nevertheless, one of the significant challenges facing the MSi community is the management and interpretation of the large volumes of data generated. To address this, considerable efforts are being made to develop innovative software solutions that enhance data processing [118,119,120,121]. These advancements focus not only on accurate co-registration algorithms but also on extracting meaningful data that can be correlated with translational patient characteristics, such as disease-free survival, recurrence, or treatment response.

(4) De novo sequencing of proteins

Alternative splicing is a widespread process in eukaryotic organisms, with more than 90% of human genes producing alternatively spliced products. Aberrant splicing is linked to numerous diseases, including inherited muscular disorders like Myotonic Dystrophy type 1 and 2, underscoring the importance of detecting splice variants for a deeper understanding of complex disease mechanisms. However, traditional methods for identifying these variants focus primarily on RNA sequencing, leaving a significant gap in the analysis of protein levels [122]. A major limitation of current proteomics approaches, particularly those relying on database searches, is their dependence on existing protein sequence references. This reliance poses challenges in accurately identifying peptides in cases where the proteome is less defined or entirely unknown, such as in non-model organisms, microbial communities, or monoclonal antibodies. Even when sequences are known, factors such as splice variants and post-translational modifications can hinder the accurate identification of MS/MS spectra using conventional database-driven search algorithms [123,124].

De novo sequencing, which infers amino acid sequences directly from experimental MS/MS spectra without the need for a reference database, offers a powerful solution to these challenges. This method is particularly advantageous for identifying novel peptides and truncated proteins resulting from alternative splicing, as it circumvents the limitations of traditional database searches.

In the context of personalized medicine, de novo sequencing has high potential for efficient proteoform recognition, point mutation detection, and monoclonal antibody assembly [125]. To facilitate its sustainable adoption in clinical and research settings, it is crucial to integrate de novo sequencing into user-friendly workflows equipped with graphical user interfaces. These tools will lower the barrier to entry for end-users, enabling broader application of this powerful technique in the analysis of splice variants at the protein level.

(5) Single-cell proteomics

In studying complex biological systems composed of diverse cell types, it is commonly assumed that each cell type has distinct functions, lineages, and molecular compositions. Traditional population-averaged assays often overlook cellular heterogeneity, failing to capture the functional and compositional differences that exist even among genetically identical cells. Extending proteomic analysis to single cells and other low-input samples can provide a deeper understanding of the roles various cell types play in normal physiology and disease processes. Moreover, it enables spatial mapping of tissues and detailed characterization of the cellular microenvironment [126,127].

Skeletal muscle, a key regulator of whole-body metabolism, consists of a complex mixture of fiber types [128]. Unlike other mammals, where muscles tend to have a predominant slow or fast fiber type, most human muscles contain a mix of fiber types. Traditional methods like enzyme and immunohistochemistry or biochemical analyses on isolated myofibers have only identified a limited number of proteins that are differentially expressed among these fiber types. Aging and various diseases affect these fiber types differently, highlighting the importance of investigating proteomic changes in a fiber-type-specific manner.

Significant progress has been made with the advent of single-fiber proteomics, allowing for a more detailed exploration of muscle fiber molecular heterogeneity [2,129]. Recent advances in proteomics, which now achieve the sensitivity needed to analyze mononucleated single cells, combined with AI-driven image analysis and laser capture microdissection, promise to further enhance our understanding of skeletal muscle diversity and plasticity [130].

However, a key challenge in single-cell proteomics remains that, unlike DNA or RNA, proteins cannot be amplified. This limitation imposes a meticulous optimization of every step of the analytical process to maximize protein detection and achieve a comprehensive view of protein expression, ideally encompassing thousands of proteins per cell. These optimization efforts span the entire proteomics workflow, from cell isolation and sample preparation to MS measurement and data processing. The combination of these technological advancements has made single-cell proteomics possible. Moreover, it is important to note that many pathophysiological processes result from a complex molecular interplay among different cell types present in the muscle tissue and that essential information might be missed by focusing only on one cell/fiber type. As such, single-cell proteomics is not only a technological breakthrough but also a necessary approach for capturing the full complexity of muscle biology.

(6) Utilization of white blood cells

Muscle biopsy sampling is an invasive procedure, highlighting the need for alternative biomaterials that can be collected through a minimally invasive process. In the last few decades, liquid biopsies have been widely used in muscle disease studies, as illustrated in Figure 1. Most research in this area has focused primarily on the study of biomarkers in plasma or serum. However, studies have shown that proteins relevant to muscle disease manifestation are expressed in white blood cells [131,132], suggesting that these cells also hold the potential to serve as minimally invasive biomaterial in the diagnosis and research of muscle diseases. In fact, previous proteomic studies on white blood cells have provided insights into the molecular etiology of various genetic muscle diseases, such as Marinesco-Sjögren syndrome [133], axonal neuropathies [134], MICU1-deficiency [135], and PTPN11-related perturbed neuromuscular transmission [136]. In addition, proteomic studies on white blood cells from patients with neuromuscular diseases have been shown to facilitate the biochemical evaluation of the pathogenicity of VUS at the biochemical level [137,138]. Accordingly, it is likely that white blood cells will increasingly become a common alternative to invasive muscle biopsies, which will serve as the basis for diagnostic and research-oriented proteomic studies. Along this line, the definition of a catalog of proteins that are of neuromuscular relevance and are expressed in white blood cells would be an essential starting point for determining which diseases can be examined in this cellular system. In the context of clinical studies aiming to restore the protein affected by the genetic defect through gene therapy approaches, targeted proteomics could allow for precise quantification of the restored (exogenous) protein, enabling correlations between its abundance and the patient’s clinical improvement. Furthermore, global proteomic studies could evaluate the effect of restored protein abundance on affected pathways—all through a minimally invasive procedure. Thus, the advantages of proteomic studies on white blood cells for diagnostic, research, and clinical studies are clear and compelling.

(7) Utilization of muscle organoids

Muscle organoids are culture systems that structurally and biochemically model human myogenesis and muscle, thus steadily gaining popularity in muscle disease research. This trend is further supported by the fact that human skeletal muscle organoids can be differentiated from induced pluripotent stem cell lines [139], which can themselves be generated from dermal fibroblasts. The latter can be cultured from small skin biopsies, which are less invasive than muscle biopsies. Hence, patient-derived fibroblasts represent a promising source for generating muscle organoids, which enable the examination of disease-relevant processes at both the structural and molecular levels. This postulate is supported by the fact that 257 of 385 proteins relevant for hereditary neuromuscular diseases (137 for myopathic disorders) are already expressed in cultured human dermal fibroblasts [140]. Differentiation into muscle organoids could even significantly increase this number by initiating the expression of muscle-specific structural proteins. To demonstrate the potential of mass spectrometry-based studies for exploring the molecular etiology of muscle diseases in muscle organoids and provide a list of hereditary muscle diseases that can be analyzed in muscle organoids based on protein coverage, we provide a catalog of neuromuscular-related proteins that are abundant in this upcoming cell culture system (see Appendix A Box 4 and Supplementary Table S1). Despite this enormous potential, such studies on muscle organoids are currently under-represented in the research of muscular diseases.

## 4. Expert Opinion

Over the last decade, mass spectrometry-based clinical proteomics has significantly improved the current understanding of myology in terms of both physiological processes underlying muscle function and pathophysiological processes leading to the clinical manifestation of a diversity of (hereditary) muscle diseases. For both sub-disciplines, unbiased proteomic profiling enabled us to obtain fundamental insights into complex biochemistry. From the diagnostic point of view, proteomic profiling offers a great opportunity to monitor the abundance of thousands of proteins by making use of a minimal amount of biopsy material. This also enables the evaluation of the pathogenicity of VUS. This advantage is not only warranted by the potential dysregulation of the protein encoded by the gene carrying the genetic defect but also by the potential impact of a dysfunctional or missing protein on pathways controlled by this protein. In the future, targeted proteomics may allow to replace the immunological-based protein profiling studies, which are still part of routine diagnostic work-up of muscle biopsies in many laboratories. A diversity of examples highlighted that proteomics even holds the potential to pinpoint the underlying genetic defect. On a general note, the discovery of pathomechanisms underlying muscular diseases is an important prerequisite in the definition of starting points for new and/or alternative therapeutic intervention concepts. Grouping different muscular diseases according to common pathophysiologies unveiled by proteomics might allow determining common treatment concepts in terms of so-called “basket trials”.

Despite these advantages, muscle proteomics still harbors limitations and challenges in the study of muscular diseases including the analyses of patient-derived biopsies per se with impacts on post-translational protein modifications, a quenched dynamic range caused by highly abundant structural proteins of skeletal musculature, quantification of different protein isoforms (which may arise from pathophysiologies), and missing proteins.

Biomarker studies are a main area of research on muscle diseases and have become an important tool in patient stratification and monitoring of clinical trials. Here, proteomics allowed the unbiased identification of a diversity of these marker proteins in the blood (serum/plasma) and CSF of patients. In the context of blood biomarkers, different studies highlighted the suitability of white blood cells to determine minimal-invasive but cellular biomarkers of direct pathophysiological impact. Combined proteomic studies on both serum/plasma and white blood cells might introduce liquid biopsies as a vulnerable tool in diagnostics and research of muscular diseases in the future. The combination of sophisticated techniques such as laser capture microdissection of structures of interest or the development of muscle organoids with mass spectrometry-based protein quantification has greatly improved the current understanding of disease processes. Protein profiling studies of white blood cells and muscle organoids allowed the introduction of suitable in vitro models providing alternatives to the more invasive muscle biopsies.

## 5. Conclusions

Mass spectrometry (MS) remains a cornerstone in contemporary clinical proteomics including myology and myopathology, offering unparalleled specificity and sensitivity in the identification and quantification of biomolecules. In this context, MS has the potential to resolve complex diagnostic challenges typically requiring genetic testing, thereby streamlining patient care. However, despite the advantages and future perspectives outlined here, the widespread adoption of MS in clinical settings for diagnosing and researching muscle diseases still faces several obstacles. These include high capital costs, the need for specialized personnel, a lack of automation, and the absence of standardized workflows. Additionally, the integration of MS instruments with laboratory information systems remains limited, and regulatory hurdles persist. Addressing these operational challenges, such as optimizing turnaround times, standardizing workflows, and developing robust bio-computational data analysis tools and storage solutions, will be critical for the future success of MS in the diagnostic workup of muscle diseases. As advancements in MS technology continue to unfold, its role in personalized medicine, therapeutic drug monitoring, and various screening programs is expected to grow, ultimately benefiting future generations with more precise and efficient healthcare solutions.

## Figures and Tables

**Figure A1:**
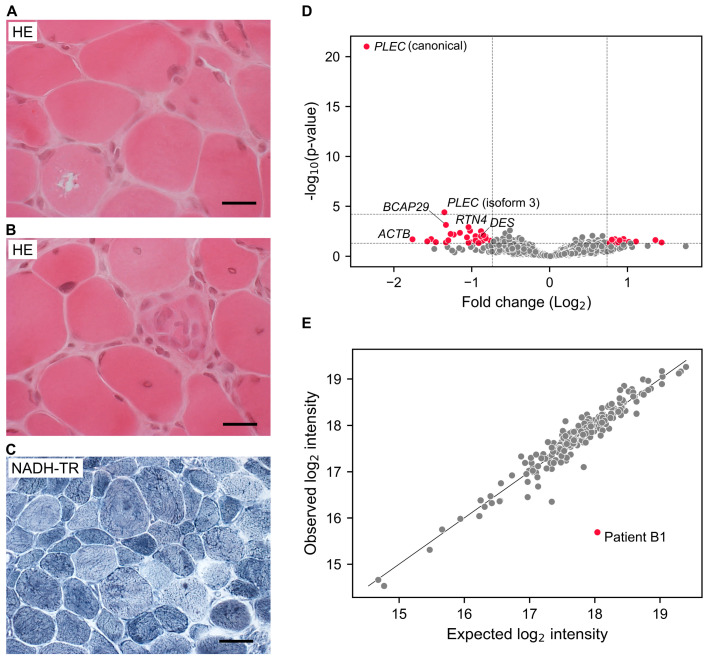
Histology and proteomics results of the case example presented in box 1. (**A**,**B**) Hematoxylin-eosin stain of patient-derived muscle biopsy specimen shows myopathological features including a rimmed vacuole and centralized myonuclei. Scale bars = 50 µm. (**C**) NADH-trichrome stain of patient-derived muscle biopsy specimen shows irregular mitochondrial distribution. Scale bar = 100 µm. (**D**) Volcano plot showing the proteomics outliers. Horizontal dotted lines represent nominal (upper line) and proteome-wide (lower line) *p*-value significance thresholds. Vertical dotted lines represent fold changes of 0.6 (left) and 1.67 (right). (**E**) Dot plot showing the expected (x-axis) versus observed (y-axis) quantity of Plectin in each individual of 216 NMD-GPS cases. Index patient is highlighted in red.

**Figure A2:**
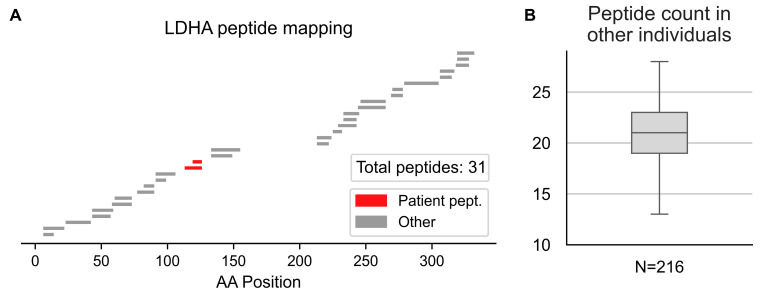
Distinct LDHA peptides identified in the proteomics experiment. (**A**)  Peptides are represented by lines showing their start and end amino acid position in the LDHA protein sequence (x-axis). In box 2 patient, only two peptides were found, shown in red. (**B**) Boxplot showing the distribution of the number of distinct LDHA peptides found in other individuals (N = 216).

**Figure A3:**
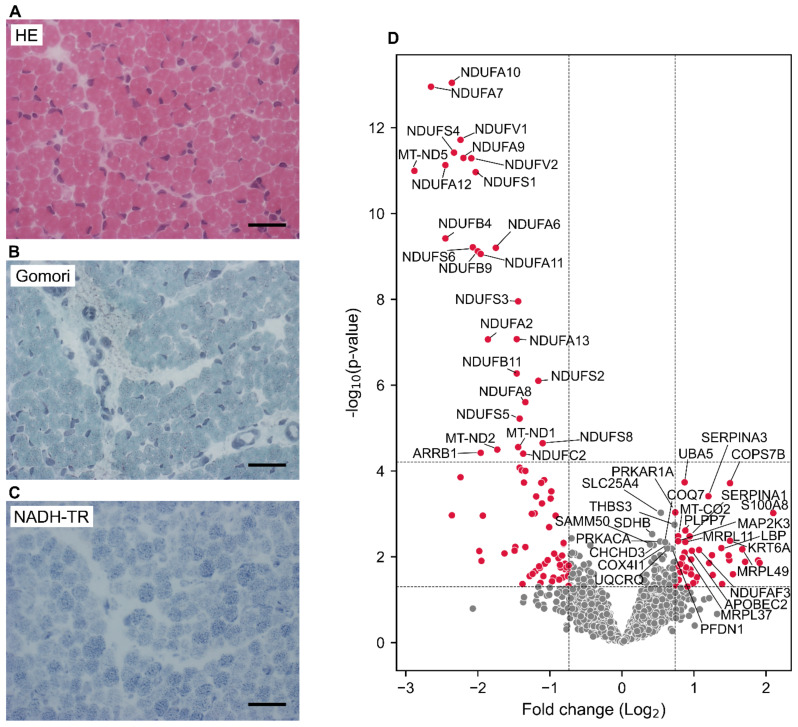
Histology and proteomics results of the case example presented in box 3. (**A**) Hematoxylin-eosin stain of patient-derived muscle biopsy specimen shows reduced muscle fiber caliber. (**B**) The gomori-trichrome stain of the patient-derived muscle biopsy specimen shows no indications of mitochondrial disease in terms of ragged red fibers. (**C**) The NADH-trichrome stain of the patient-derived muscle biopsy specimen does not reveal significant indications of abnormal mitochondrial distribution and/or activity. Scale bars = 75 µm. (**D**) Volcano plot showing the proteomics outliers of the patient presented in this box. Horizontal dotted lines represent nominal and proteome-wide significance thresholds. Vertical dotted lines represent fold changes 0.6 and 1.67.

## Data Availability

Proteomic data presented in this study are available upon request.

## Supplementary Materials

The following supporting information can be downloaded at: https://www.mdpi.com/article/10.3390/biom15010130/s1, Table S1: List of neuromuscular relevant proteins expressed in muscle organoids compared to a DIA spectral library of human skeletal muscle.

## Appendix A

**Box 1:**

Patient: A female patient of German origin was seen at the age of 22 years in the neuromuscular ambulance of the University Hospital Essen (Germany). She presented with proximal muscle weakness and atrophy and elevated creatine kinase level (>1200 U/L), recurrent rhabdomyolysis, contractures, and cardiomyopathy in addition to ptosis and learning difficulties. Quadriceps muscle biopsy showed occasionally the presence of rimmed vacuoles (Figure A1A) and internalized myonuclei (Figure A1B). The NADH-trichrome stain of patient-derived muscle biopsy specimen shows irregular mitochondrial distribution (Figure A1C).

Molecular genetics: To clarify the genetic cause of the phenotype of this patient, exome sequencing was performed and unveiled three heterozygous variants in the PLEC gene (c.8167_8185dup (p.Leu2729Profs*133), c.8587G>A (p.Asp2863Asn), and c.11378G>C (p.Arg3793Pro) encoding the Plectin protein.

Proteomic profiling: Given that the (p.Asp2863Asn) variant is known to be benign but the pathogenicity of the (p.Arg3792Pro) variant is of unclear significance according to ACMG classification, in the context of the NMD-GPS project, global proteomic profiling was carried out in a DIA mode as described in Appendix B. Unbiased MS-based proteomic analysis in a mixed cohort of 216 individuals enrolled in NMD-GPS was performed, using DIA-NN and Iq for peptide and protein identification and quantification and PROTRIDER for detection of outlier features [141,142,143]. The analysis showed a statistically highly significant reduction (fold-change: 0.196, adjusted *p*-value: 2.3 × 10^−17^) in Plectin protein levels in this patient, demonstrating that the sum of the nonsense frameshift and both missense variants lead to a drastic reduction of Plectin levels in skeletal muscle. Moreover, the reduction of Plectin may impact on Desmin, a known binding partner of Plectin, which was also found to decrease (fold-change: 0.54, nominal *p*-value: 0.01). A slight decrease was also observed in α-Actinin-3, a component of the Z-discs and interactor of Plectin although this was not statistically significant (fold-change: 0.42, *p*-value: 0.27) [144]. Several other cytoskeletal protein subunits were found up- or down-regulated at varying degrees. Proteomic findings are shown in Figure A1D,E of box 1.

Conclusions: The strong decrease in Plectin levels could be explained by the pathogenicity of the (p.Arg3792Pro) missense variant (in addition to the nonsense variant). As Plectin plays a structural role in muscle fibers localizing in the periphery of Z-discs and linking them with the surrounding Desmin intermediate filaments, the decrease in Desmin intermediate filaments most likely represents a consequence of the loss of Plectin [145]. A similar finding has also been described by Winter and colleagues [144]. Overall, this example highlights the utility of DIA proteomics to elucidate the impact of VUS. Given that PLEC is a large-sized protein, a bottom-up proteomic profiling approach might be superior to Western blotting for robust quantification. Here, unbiased proteomic profiling even enables the detection of the concomitant dysregulation of PLEC binding partners, thus highlighting another benefit of proteomics as a promising analytical approach for “protein first” strategies in diagnostic testing of patients presenting with muscular diseases.

**Box 2:**

Patient: A 19-year-old male patient of North African origin (consanguineous parents) was seen at the neuromuscular service of the Institute of Myology Paris (France). He presented from infancy with episodes of muscle pain, contractures, high creatine kinase levels (up to 6000 U/l), and one episode of rhabdomyolysis. The electromyogram showed a diffuse pseudo-neurogenic pattern with chronic myogenic damage. Muscle biopsy showed fiber-side variants and atrophic fibers as well as occasionally subsarolemmal vacuoles immunoreactive for L-lactate dehydrogenase A chain (LDHA).

Molecular genetics: To clarify the genetic cause of the phenotype of this patient, exome sequencing was performed and revealed a homozygous frameshift deletion in exon 7 (c.766_767delGT, p.Val256Serfs*12) of the *LDHA* gene (Hentschel et al., 2024 [146]).

Proteomic profiling: To investigate the effect of this frameshift variant on the global proteomic signature and to examine if a truncated protein is expressed as suggested by our immunostaining findings, proteomic profiling in DIA mode (see Appendix B) was carried out enabling the identification of 254 dysregulated proteins, including LDHA. Mapping of precursor peptides on the protein sequence indeed indicated the expression of a truncated protein compared to controls, where identified unique peptides span the entire amino acid sequence of LDHA (Figure A2).

Conclusions: The proteomic data, in combination with immunostaining results, is indicative of the expression of a truncated protein with a toxic gain of function, most likely forming toxic aggregates (Hentschel et al., 2024 [146]).

**Box 3:**

Patient: A 2-month-old male patient of German origin with the clinical presentation of mitochondriopathy (Leigh syndrome) with failure to thrive, circulatory arrest, altered basal ganglia, and elevated serum lactate level. Quadriceps muscle biopsy showed reduced muscle fiber caliber, but no pathological changes strongly indicative of the presence of a mitochondriopathy (Figure A3A–C).

Proteomic profiling: Proteomic profiling on protein extract of the quadriceps biopsy was performed in a DIA mode as described in detail in Appendix B to investigate if a mitochondrial defect, fitting with the phenotype, is indeed present in the skeletal muscle of this patient. Peptide and protein identification and quantification were done with DIA-NN and Iq, and the identification of outliers was done with PROTRIDER. This approach revealed the significant dysregulation of 38 proteins of complex I of the mitochondrial respiratory chain, raising suspicion of a primary defect in a gene that encodes a component of this complex (Figure A3D).

Molecular genetics: To further clarify the genetic cause of the phenotype of this patient in terms of a mitochondriopathy based on a complex I defect, exome sequencing was performed and showed two compound heterozygous missense variants in the NDUFA10 gene (p.Arg130His & p.His329dup), which have not yet been described in the literature as causing disease.

Conclusions: Bi-allelic NDUFA10 variants are known to lead to mitochondrial complex I deficiency, nuclear type 22 (MC1DN22) [147]. Accordingly, the proteome not only provided an indication of the underlying genetic defect but also a clear indication of the pathogenicity of the identified variants.

**Box 4:**

Biomaterial: Skeletal muscle organoids (SMOs) were generated by casting human pluripotent stem cells into a collagen I hydrogel. Utilizing a directed differentiation protocol, cells were guided through prototypical stages of muscle development [148,149]. To facilitate muscle maturation, SMOs were subjected to mechanical loading resulting in a contractile three-dimensional muscle for functional phenotyping.

Proteomic profiling: Proteomic profiling on protein extract of the biological replicates of SMOs was performed in DIA mode as described in detail in Appendix B.

Conclusions: Mass spectrometry-based protein profiling on healthy SMOs allowed the cataloging of 5735 proteins (see Supplementary Table S1). These proteins cover 41% of the proteins attributed to the manifestation of subtypes of congenital myasthenic syndromes (CMS; Figure A4), based on pathogenic variants in the corresponding genes, as well as 84% and 69% of proteins responsible for the manifestation of spinal muscular atrophy and myopathies, respectively (for more details see Supplementary Table S1). This protein catalog may aid in the selection of neuromuscular diseases that are suitable for analysis in human SMOs. It can serve both diagnostic purposes, such as evaluating the pathogenicity of gene variants for which the corresponding protein is exclusively expressed in skeletal muscle cells, and research purposes.

## Appendix B

(1) Sample preparation for mass spectrometry

Samples that had been snap-frozen were subjected to lysis by the addition of 200 µL of 50 mM triethylammonium bicarbonate (TEAB) buffer (pH 8.5), containing 5% sodium dodecyl sulfate (SDS) and a protease inhibitor cocktail (cOmplete ULTRA, Roche, Basel, Switzerland). The lysed samples were then processed using the Bioruptor^®^ sonication system (Diagenode, Denville Township, NJ, USA) under the following conditions: 10 min of treatment at 4 °C with alternating 30-s cycles of sonication (on and off), for a total of 10 cycles. To ensure complete lysis, we conducted an additional sonication step using an ultra-sonic probe (30 s, 1 s/1 s, amplitude 40%) followed by centrifugation at 4 °C and 20,000× *g* for 15 min to remove debris. Protein concentration in the supernatant was quantified using a bicinchoninic acid (BCA) assay, following the manufacturer’s instructions.

To reduce disulfide bonds, tris(2-carboxyethyl)phosphine (TCEP) was added to a final concentration of 10 mM, and the reaction was incubated at 37 °C for 30 min. Subsequently, free sulfhydryl groups were alkylated by the addition of 15 mM iodoacetamide (IAA) and incubated at room temperature in the dark for 30 min.

For proteolytic digestion, 100 µg of protein from each sample was processed according to the S-Trap protocol (Protifi, Fairport, NY, USA), with a protein-to-trypsin ratio of 20:1. The samples were incubated for proteolysis at 47 °C for 2 h.

Following digestion, the resulting peptide mixtures were desalted according to manufacture instructions (Protifi, Fairport, NY, USA), and digestion completeness was verified by monolithic column separation using a PepSwift PS-DVB (PL-CAP200-PM, Dionex, Germering, Germany) column on an Ultimate 3000 HPLC system (Dionex, Germering, Germany). A 0.5 µg aliquot of each sample was directly injected into the column. A binary gradient was employed for separation, with solvent A consisting of 0.1% trifluoroacetic acid (TFA) in water, and solvent B composed of 0.08% TFA in 84% acetonitrile (ACN). The gradient progressed from 5% to 12% solvent B over 5 min, followed by an increase to 50% solvent B over 15 min, at a flow rate of 2.2 µL/min and a temperature of 60 °C. UV absorbance was monitored at 214 nm to confirm peptide separation [150].

(2) Mass spectrometry

All samples were analyzed using an UltiMate 3000 RSLC nano UHPLC system (ThermoFisher Scientific, Bremen, German) coupled with a QExactive HF mass spectrometer. For each analysis, a total of 1 µg of peptides was injected. Initially, the peptide mixtures were loaded onto a pre-column (75 µm × 2 cm, 100 Å, C18) at a flow rate of 10 µL/min for 20 min. The peptides were then separated on the analytical column (75 µm × 50 cm, 100 Å, C18) with a flow rate of 250 nL/min. The separation was performed using a linear gradient, where mobile phase A consisted of 99.9% water and 0.1% formic acid, and mobile phase B consisted of 84% acetonitrile, 15.9% water, and 0.1% formic acid. The gradient spanned 120 min, progressing from 3% to 35% of solution B. Specifically, the gradient steps were as follows: 3% B for the first 20 min, followed by an increase from 3% to 35% B over 120 min. After the gradient, three wash steps were conducted, each bringing the concentration of buffer B to 95% for 3 min. Finally, the system was allowed to equilibrate for 20 min before the next sample was introduced.

Mass spectrometry (MS) data were acquired using data-independent acquisition (DIA) mode, supported by an in-house generated spectral library. Each sample was spiked with an appropriate amount of iRT (indexed retention time) standard (Biognosys, Schlieren, Switzerland) to ensure accurate retention time alignment. Full MS scans were conducted within the mass-to-charge (*m*/*z*) range of 300–1100, using the Orbitrap mass analyzer at a resolution of 60,000. The polysiloxane ion at 445.12002 *m*/*z* served as the lock mass for internal calibration. The automatic gain control (AGC) was set to a target value of 3 × 10^6^, with a maximum injection time of 20 milliseconds. Following each full MS scan, 23 DIA windows were acquired, each spanning 28 *m*/*z* with a 1 *m*/*z* overlap, starting at 400 *m*/*z*. These DIA scans were collected with a resolution of 30,000 on the Orbitrap, using an AGC target of 3 × 10^6^ and a normalized collision energy (nCE) of 27 for fragmentation via collision-induced dissociation (CID).

(3) Mass spectrometry data analysis

For the analysis of samples acquired via nano-LC-MS/MS in data-independent acquisition (DIA) mode, the raw data were processed using the Spectronaut software (version 18) (Biognosys) with a directDIA approach, utilizing a spectral library in addition. The spectral libraries employed were generated in-house and tailored to the specific sample type being studied. Default settings from the Biognosys factory (BGS) configuration were applied for both the search and data extraction parameters. The human proteome database from UniProt (UniProtKB ID: UP000005640_9606) [151], containing 20,374 entries, was used as the reference background for protein identification.

To ensure robust label-free quantification, only proteins identified by at least two unique peptides were included in subsequent analyses. Normalized abundance values, calculated within Spectronaut, were averaged for each protein across replicates. These normalized averages were then used to determine the abundance ratios between the different sample types and their respective control groups.

The statistical significance of changes in protein abundance was assessed using the Student’s *t*-test. This analysis was performed for each protein using Microsoft Excel, and the corresponding *p*-values were calculated to evaluate the significance of observed differences.

(4) Mass spectrometry data analysis—NMD-GPS cohort

The raw proteomics files were converted to mzML format using the ThermoRawFileParser (version 1.4.2) [152]. Peptide identification and quantification was done with DIA-NN (version 1.8.1) using library-free search and match-between-runs enabled, for all 648 samples (216 triplicates) of the NME-GPS cohort [141]. For the first step of the library-free search, in silico prediction of the spectral library was done from the full human proteome reference and additional sequences obtained from UniProt (UniProtKB ID: UP000005640_9606) [151]. The false discovery rate threshold was set to 1% for peptide and protein identification. The R package iq (version 1.9.12) was used for protein quantification, which implements the MaxLFQ algorithm for DIA-based data [142,153]. Finally, protein intensity values were log-transformed. The statistical significance of changes in protein abundance was assessed with PROTRIDER [143].
